# The Accuracy of Praziquantel Dose Poles for Mass Treatment of Schistosomiasis in School Girls in KwaZulu-Natal, South Africa

**DOI:** 10.1371/journal.pntd.0004623

**Published:** 2016-05-03

**Authors:** Marije Baan, Hashini Nilushika Galappaththi-Arachchige, Silindile Gagai, Christine G. Aurlund, Birgitte J. Vennervald, Myra Taylor, Lisette van Lieshout, Eyrun F. Kjetland

**Affiliations:** 1 Department of Parasitology, Leiden University Medical Centre, Leiden, The Netherlands; 2 Faculty of Health, Medicine and Life Science, Maastricht University, Maastricht, The Netherlands; 3 Norwegian Centre for Imported and Tropical Diseases, Department of Infectious Diseases, Oslo University Hospital, Oslo, Norway; 4 Faculty of Medicine, University of Oslo, Oslo, Norway; 5 Discipline of Public Health Medicine, Nelson R Mandela School of Medicine, College of Health Sciences, University of KwaZulu-Natal, Durban, South Africa; 6 Section for Parasitology and Aquatic Diseases, Faculty of Health and Medical Sciences, University of Copenhagen, Copenhagen, Denmark; Children's National Medical Center, UNITED STATES

## Abstract

**Background:**

More than 260 million people live with schistosomiasis and regular mass-treatment should be implemented to prevent morbidity. Praziquantel, dosed at 40 milligrams per kilogram bodyweight, is the drug of choice. During the last decades the WHO Tablet Pole–which estimates tablet need by height as representing weight–has been used as a practical and cheap tool in mass treatment. In South Africa this method could be inaccurate given the prevalence of overweight and obesity. In this study in female pupils in KwaZulu-Natal, South Africa, we explored the accuracy of the WHO Tablet Pole and the recently developed Modified Dose Pole for adults with two additional intervals and correction for body mass index (BMI).

**Methodology:**

In randomly selected primary and secondary schools of schistosomiasis-endemic areas, height and weight of female pupils were measured. The WHO Tablet Pole and Modified Dose Pole were used to indicate the amount of praziquantel according to height and the dose in milligrams per kilogram bodyweight was calculated. The BMI correction was performed by adding 600 milligrams (1 tablet) to the indicated dose if a person was overweight/obese.

**Principal Findings:**

3157 female students were investigated and 35% were found to be overweight/obese. Using the WHO Tablet Pole, 73% would have received an adequate dose (range 30–60 mg/kg). When correcting for BMI, this would have been 94%. Using the Modified Dose Pole with BMI correction, 97% would have been adequately treated.

**Conclusions:**

This study shows that the WHO Tablet Pole will be inaccurate in estimating the dose of praziquantel in South African girls due to high prevalence of overweight/obesity. Under-dosing of individuals who appear overweight/obese could be largely prevented by adding an extra praziquantel tablet to the recommended dose. Further research must be done to explore if subjective weight estimates are reliable.

## Author Summary

Schistosomiasis is an acute and chronic parasitic disease caused by *Schistosoma* worms, contributing to morbidity and mortality in 261 million people, mainly in poor, rural communities. In an effort to control schistosomiasis, the World Health Organization advocates for regular mass treatment to at-risk populations, administering free praziquantel (40mg/kg). The WHO Tablet Pole, which estimates the number of tablets for each individual by height, is recommended for the dosing of praziquantel. A Modified Dose Pole with two additional height intervals has been developed for adults. In addition, the developers of the Modified Dose Pole suggest to add an extra tablet to the indicated dose if the person appears to be overweight/obese (“correction for body mass index (BMI)”). This study explored the accuracy of these dose pole programmes in 3157 school-girls in schistosomiasis-endemic KwaZulu-Natal, South Africa. Overweight or obesity was found in 35% of the study population. With the WHO Tablet Pole, only 73% would have received an acceptable dose (30-60mg/kg), whereas when correcting for overweight/obesity almost all the girls would have been adequately treated. We suggest adding one tablet to the dose recommended by the WHO Tablet Pole if a person appears to be overweight/obese to prevent under-dosing of these individuals.

## Introduction

Schistosomiasis remains an important health challenge in poor, rural communities, contributing to significant morbidity and mortality [1,2]. As a means to control schistosomiasis, the World Health Organization (WHO) advocates for regular treatment with praziquantel (PZQ) of at-risk populations, an intervention that has been shown to be safe and cost-effective [3,4]. South Africa, particularly in the Eastern and Northern parts of the country, is endemic for schistosomiasis and an estimated 2.4 million school-aged children and 2.7 million adults require treatment with praziquantel yearly or every second year [3,5].

Though often times neglected, schistosomiasis has recently gained attention as several studies suggest that it may increase the risk of HIV infection in females [6–10]. The prevalence of HIV/AIDS in South Africa is among the highest in the world [11] and it has been proposed that regular mass-treatment of schistosomiasis could both reduce the burden of disease and have an impact on HIV transmission rates [12,13].

Currently, South Africa has no national schistosomiasis control programme. However, the South African Integrated School Health Policy (ISHP) has adopted a policy to offer regular treatment to children attending schools in schistosomiasis-endemic areas [5,14]. Between the years 1997 and 2000 a helminth-control programme (including schistosomiasis treatment) was piloted in KwaZulu-Natal, revealing the necessity and efficacy of regular treatment of schistosomiasis [15].

Treating schistosomiasis requires a praziquantel dose that is adjusted to bodyweight; 40 milligrams per kilogram (mg/kg) [16]. The provision and maintenance of weight scales to the endemic schools that are mostly remote and located in rural areas may constitute a major impediment for implementation of school-based programmes. Hall et al. therefore investigated an alternative for bodyweight and found that in children from Tanzania, Ghana and Malawi, height roughly corresponded to bodyweight [17]. In 2001, the WHO Tablet Pole–which estimates the dose of praziquantel by height–was developed and this pole has been recommended for mass-treatment of school-aged children [18,19]. A revision of this dose pole which allows for inclusion of preschool-aged children was proposed by Sousa-Figueiredo et al.[20]. Though the WHO Tablet Pole has been validated in multiple populations, recent studies have discussed its reduced accuracy in groups where height and bodyweight are less correlative [21–23]. Palha De Sousa et al. have shown that in adults, overweight or obese individuals are likely to receive an insufficient dose when using the WHO Tablet Pole [23]. Therefore, a Modified Dose Pole–which incorporates a correction for body mass index (BMI)–was developed for use in adults [23].

South Africa has the highest prevalence of childhood obesity of sub-Saharan Africa; 19% of boys and 26% of girls below the age of 20 years are estimated to be overweight or obese [24]. Yet the accuracy of the WHO Tablet Pole in this population remains undetermined. In light of the pending treatment programmes in South Africa, this study sought to explore the accuracy of dosing by height and the necessity to correct for overweight/obesity in girls of primary and secondary schools in KwaZulu-Natal.

## Methods

### Study design and participants

This cross-sectional study made use of two data sets originating from on-going studies on schistosomiasis in girls and young women in KwaZulu-Natal. The first data set was collected in 18 randomly selected primary schools (2009–2010) and included girls between 10 and 12 years of age. The second data set was collected in 70 randomly selected secondary schools (2011–2013) and included adolescent girls, aged 16 years and above. Selection of the schools was conducted through systematic sampling from all large and medium size schools in the districts and all girls within the target population were invited to participate in the study.

For both cohorts, exclusion criteria were absence on the invitation days, serious illness or lack of consent. For the present study, all individuals from the original study population (n = 3419) who were lacking data on height, weight or age were excluded (n = 206). Furthermore, of the secondary school cohort, all pupils above the age of 23 years were excluded (n = 56).

Participants originated from the coastal districts of KwaZulu-Natal; Ugu, Ilembe, and Uthungulu, which are all endemic for *S*. *haematobium* [5]. KwaZulu-Natal is one of the nine provinces of South Africa and has approximately 10.3 million inhabitants, of which more than half reside in rural areas [25]. An average of 56.6% of the households in KwaZulu-Natal live below national poverty lines and 14.1% have no access to piped water [26,27]. Furthermore, even if they have access to potable water, many continue to use the river and dams for recreational purposes, laundry and bathing.

### Ethical considerations

This study drew from a larger project that was approved by the Biomedical Research Ethics Administration, University of KwaZulu-Natal (BREC, 2009, Ref BF029/07), the Departments of Health and Education of KwaZulu Natal (2009, Ref HRKM010-08), The Norwegian Ethics Committee, Eastern Norway (REC, 2007, Ref IRB 0000 1870, amended 2011), The European Group on Ethics in Science and New Technologies (2011, Ref IRSES-2010:269245), and the Departments of Health (2008) and Education in Ugu (2009), Ilembe (2012) and Uthungulu (2013).

Information sessions were held at each school before invitation to participate in the study. All participants signed individual informed consent forms. Assenting participants younger than 12 years of age were only included if a consent form was signed by their guardian. The ethical committees, BREC (annual renewal) and REC, specifically approved the consent procedures for adolescents below the age of 18 years (independent minor consent, no parental consent) [9]. School-based treatment was offered to all students in the included schools by the Districts’ Departments of Health.

### Data collection

One to three urine samples were collected and analysed for the presence of *Schistosoma* eggs as a part of the nested cohort study. For comparability reasons, only the first urine sample was taken into account in the present study. An individual was considered to be schistosomiasis positive if at least one terminal spined ovum was seen in the urine sample.

Height was measured up to a precision of 1 cm, using a stadiometer. Weight was measured up to a precision of 0.1 kg, using an electronic scale. Each individual was classified as underweight, normal weight, overweight or obese, based on the calculated body mass index (BMI, height/weight^2^). In adults (18 years of age and above) underweight, normal weight, overweight, and obese were defined as a BMI less than 18.5, from 18.5 to 25, from 25 to 30, and more than 30, respectively. In individuals under the age of 18 years, the BMI classification was based on the International Obesity Task Force cut-off points (IOTF) (S1 Table) [24,28].

### Data analysis

**Fig 1:**
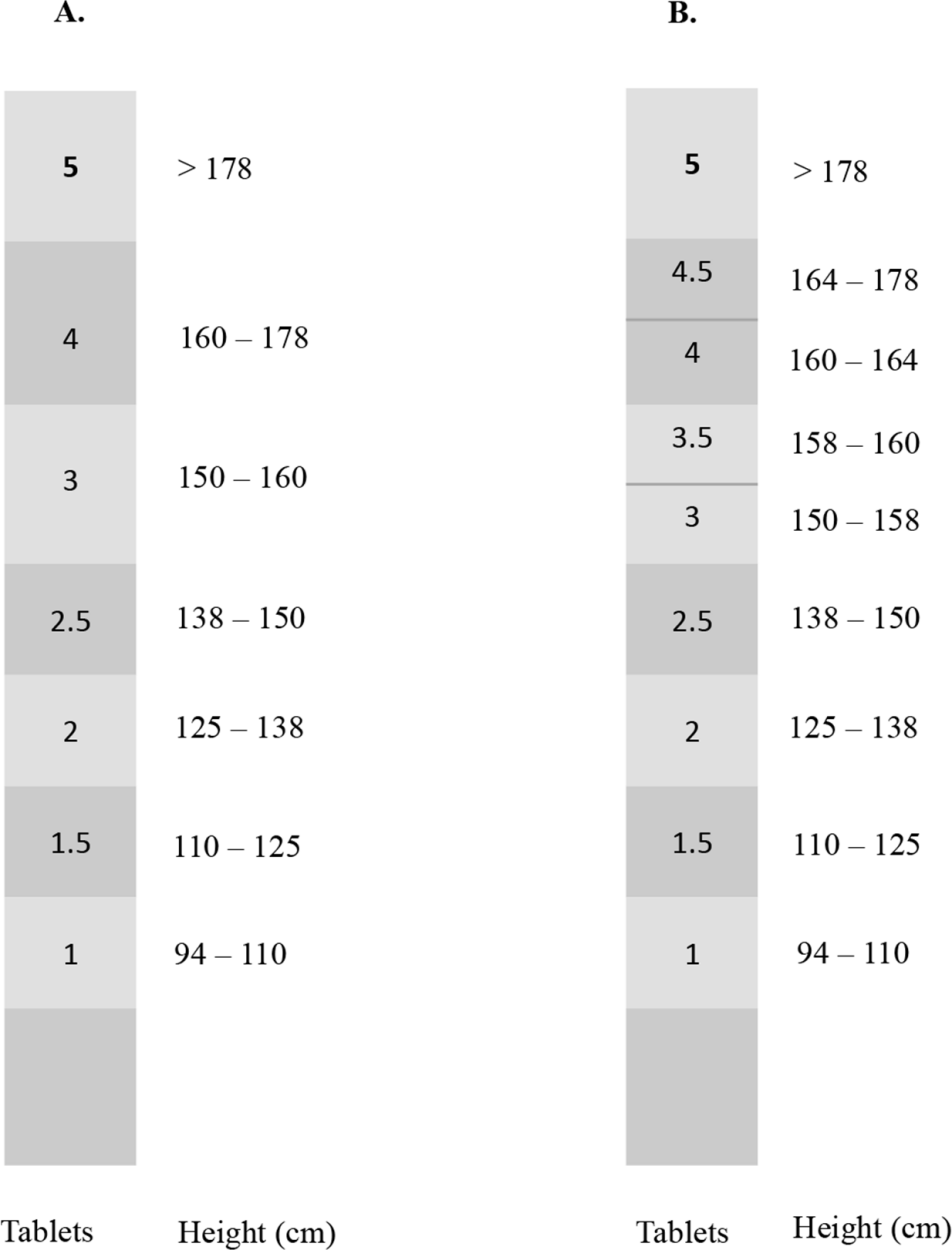
**The WHO Tablet Pole (A) and the Modified Dose Pole (B)**. The WHO Tablet Pole (A) and the Modified Dose Pole (B) have 7 and 9 height intervals respectively that indicate the corresponding dose of praziquantel in tablets of 600 mg each. BMI correction can be performed by adding one tablet of praziquantel for overweight and obese patients. The use of pictograms has been proposed to simplify BMI classification in practice [23].

The WHO Tablet Pole and the Modified Dose Pole are presented in Fig 1. The accuracy of the dose pole programmes in indicating a dose of 40mg/kg was investigated by calculating the dose in mg/kg that would have been assigned to each individual in the study population. Based on their height, individuals were classified in one interval of each dose pole. The indicated dose in milligrams (mg) was then divided by the bodyweight of the individual. The correction for BMI was performed by adding one 600 mg tablet to the total dose if an individual was classified as overweight or obese.

The optimal dose was defined as receiving 40 to 60 mg praziquantel per kilogram bodyweight; receiving less than 30 mg/kg was defined as an insufficient dose and an excessive dose was defined as receiving more than 60 mg/kg. A dose between 30 and 60 mg praziquantel per kilogram was considered to be an appropriate dose [16,18,29].

Statistical analyses were performed using IBM SPSS statistics version 22. Chi-square was used to compare the prevalence of overweight and obesity and schistosomiasis between the primary and secondary school cohort. Multivariate Odds Ratio (OR) was used to investigate an association between receiving an insufficient dose and being overweight or obese. For the percentages of the population within each dosing category or BMI classification, 95% Confidence Intervals (CI) were given.

## Results

### Group characteristics

The study population included 3157 female students of which 1008 were in primary schools and 2149 in secondary schools. The median age of the primary school students was 11 years (range 10–12); in the secondary school students the median age was 18 years (range 16–23) (S1 Dataset). In the students for whom a urine sample was investigated (n = 2968), the prevalence of *S*. *haematobium* infection was on average 22.6% (CI 21.1–24.1). The prevalence of infection was significantly higher in primary schools (32.2%, 302/938, CI 29.3–35.3), as compared to secondary schools (18.2%, 369/2030, CI 16.5–19.8, p<0.01).

### BMI distribution

Overweight or obesity was found in 34.5% (CI 32.9–36.2) of the study population. It was more common in secondary school students (40.9%, CI 38.5–43.0) than in primary school students (21.0%, CI 18.6–23.5). The prevalence of underweight was 4.5% (CI 3.8–5.2). The prevalence of obesity was 6.3% (CI 3.6–9.2) in the youngest age group (10 years of age) and 14.5% (CI 5.5–23.6) in the oldest age group (23 years of age). This difference was not significant, however the prevalence of overweight was significantly higher in those 23 years of age (41.8%, CI 23.3–54.5) compared to those 10 years of age (15.5%, CI 11.5–20.1, p<0.01). Schistosomiasis infection was found in 32.0% (CI 25.9–38.1) of the overweight/obese students in primary schools and in 15.6% (CI 13.1–18.2) of the overweight/obese students in secondary schools. The prevalence of *S*. *haematobium* was not significantly different in overweight/obese individuals compared to those with a normal BMI, when corrected for age.

### Dosing based on height

**Fig 2:**
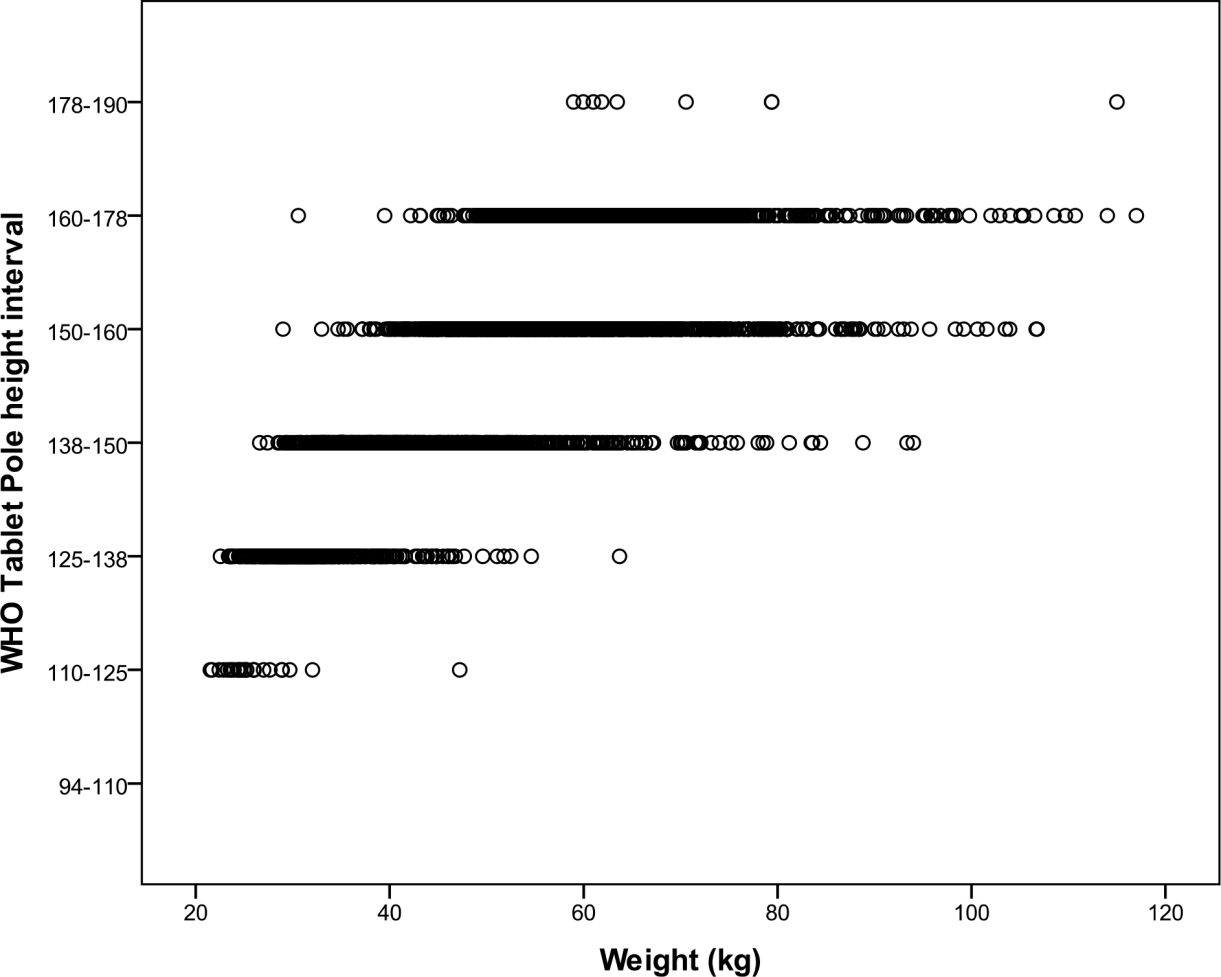
The weight range for the WHO Tablet Pole height intervals. All individuals in one interval would have received the same number of praziquantel tablets.

In our study population we found a large variation of bodyweight, which can only partly be explained by the variation in height (r^2^ = 0.55). For the height intervals in the WHO Tablet Pole, a large weight range was observed for each interval, as shown in Fig 2. The same dose would have been indicated to persons whose bodyweights varied on average 60 kg per dose interval. The weight range was largest (31–117 kg) in the 160–178 cm height interval (corresponding with 4 tablets).

The dosages that would have been provided using the WHO Tablet Pole and the Modified Dose Pole are presented in Table 1. Of the study population 73.0% (CI 71.3–74.4) would have received an appropriate dose (30 to 60 mg/kg) with the WHO Tablet Pole. In the primary schools, 89.2% (CI 87.3–91.0) would have been given a dose between 30 and 60 mg/kg compared to 65.3% (CI 63.2–67.3) in the secondary schools. Using the BMI corrected WHO Tablet Pole 93.6% (CI 92.7–94.5) of the total population would have received an appropriate dose of praziquantel. In the primary schools this was 98.6% (CI 97.8–99.3), whereas in secondary schools 91.2% (CI 90.0–92.4) would have received enough praziquantel (S2 Dataset). Almost 96.5% (CI 95.8–97.1) of the total population would have received an appropriate dose if using the BMI corrected Modified Dose Pole (p<0.001). If not correcting for BMI, the Modified Dose Pole would have resulted in 79.9% (CI 78.5–81.3) receiving an appropriate dose, a significant difference compared to the WHO Tablet Pole (p<0.001). The percentage that would have received an excessive dose (more than 60 mg/kg) was 0.1% for all dose pole programmes. Of the total population, 27.0% (CI 25.5–28.6) would have received an insufficient dose (less than 30 mg/kg) using the WHO Tablet Pole, but only 0.9% would have received doses below 20 mg/kg (data now shown). The average dose that would have been given using the WHO Tablet Pole was 34.7 mg/kg, which falls into the lower end of the appropriate range. Using the BMI corrected WHO Tablet Pole the average dose would have been 37.9 mg/kg. The average dose that would have been indicated by the Modified Dose Pole with BMI correction was 39.4 mg/kg.

**Table 1:** Dosages of praziquantel that would have been administered using the WHO Tablet Pole and the Modified Dose Pole (with and without BMI correction) in a population of 3157 female students of primary and secondary schools in South Africa.

Dosing category	WHO Tablet Pole				Modified Dose Pole	
	Without BMI correction			With BMI correction	Without BMI correction	With BMI correction
	*10–12 years* ^a^ *(n = 1008)*	*16–23 years* ^b^ *(n = 2149)*	*Total (n = 3157)*	*Total (n = 3157)*	*Total (n = 3175)*	*Total (n = 3175)*
Insufficient (<30 mg/kg)	10.7%	34.6%	27.0%	6.3%	20.0%	3.4%
Acceptable (30–40 mg/kg)	50.6%	51.4%	51.2%	61.4%	50.8%	53.1%
Optimal (40–60 mg/kg)	38.6%	13.9%	21.8%	32.2%	29.1%	43.4%
Excessive (≥60 mg/kg)	0.1%	0.1%	0.1%	0.1%	0.1%	0.1%

The acceptable and optimal dosages are defined as appropriate.

^a^Primary school

^b^Secondary school

**Fig 3:**
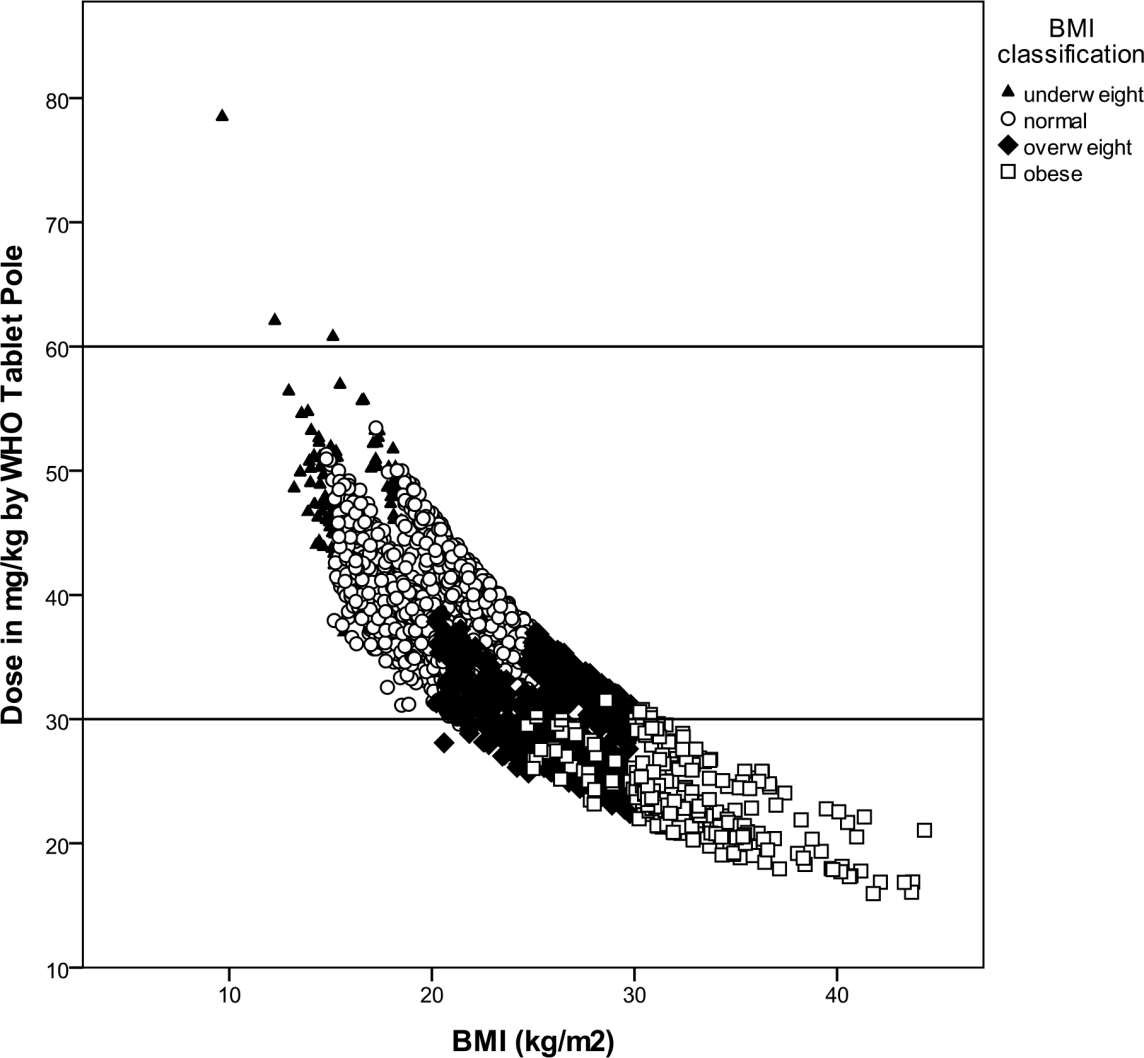
The relationship between body mass index and dose received using the WHO Tablet Pole. The increase in tablet interval (from ½ tablet to 1) at a height of 160 cm appears as a gap in the study population. The horizontal lines present the range of an appropriate praziquantel dose between 30–60 mg/kg.

The relationship between the body mass index and the dose that would have been indicated using the WHO Tablet Pole is shown in Fig 3. Being overweight or obese was associated with receiving an insufficient dose (OR 38.5, CI 30.5–48.5). Of all cases that would have received an insufficient dose, 55.9% were overweight and 31.3% were obese. Of the individuals that were classified as obese, 97.1% would have received an insufficient dose using the WHO Tablet Pole. The maximum dosage using the WHO Tablet Pole is 3000 mg of praziquantel (for the height interval 178–190 cm), but this dose would only be sufficient for bodyweights up to 75 kg (assuming a praziquantel dose of 40 mg/kg). Of the study population, 109 individuals (3.5%) weighed more and would have required more praziquantel.

Simulating a dose pole designed for praziquantel dosing at 60 mg/kg rather than the standard 40mg/kg (by adding 50% in each of the height intervals), 77.2% (CI 75.8–78.7) of the population would have received a dose between 30 and 60 mg/kg, while 21.9% (CI 20.4–23.2) would have received more than 60 mg/kg. Out of the 3157 pupils, 10 (0.3%) would have been given more than 80 mg/kg. Only 0.9% would have received a dose of less than 30 mg/kg.

### Total use of tablets

Using the WHO Tablet Pole, 9543 tablets would have been required for a population of 3157 girls; on average 3 tablets per girl. If a correction for BMI was used for the WHO Tablet Pole, 10633 tablets would be needed, which is 11% more than without this correction. The Modified Dose Pole with BMI correction required 17% more tablets (S2 Dataset). Per 1000 pupils in primary schools 210 extra tablets would be needed and per 1000 pupils in secondary schools 409 extra tablets would be needed when correcting for BMI using the WHO Tablet Pole.

### Dosing based on bodyweight

Dosing based on bodyweight requires the availability of weight scales. Relatively robust electronic bathroom scales that can weigh one thousand children repeatedly cost USD 15–20 per scale, whereas the dose pole can be printed on paper and be hung on the wall. If one scale is bought per 300 pupils they would cost USD 165 000 as a conservative estimate (Table 2). Mass-treatment is performed yearly or every second year and the scales would either have to be stored centrally or at each school. If stored centrally only 665 scales would be needed for the same number of schools, but 40 metres of shelves would be needed to store them (45 cm high, 6 cm width per scale × 665 scales). In addition, there would be a cost for distribution in a timely manner to each school. Furthermore, batteries would have to be ensured.

**Table 2:** The cost of weight scales for mass treatment in schools in the KwaZulu-Natal Province.

School size	Scales per school	Number of schools this size^a^	Number of scales needed^b^	Price per scale (USD)	Price total (USD)
Up to 300 pupils	1	1 909	1 909	15	
300–600 pupils	2	1 946	3 892	15	
600–900 pupils	3	957	2 871	15	
More than 900 pupils	3	795	2 385	15	
**Totals**		**5 607**	**11 057**	**15**	**165 855**

^**a**^In KwaZulu-Natal Province.

^**b**^If scales are left in the schools.

## Discussion

In girls of primary and secondary schools in rural KwaZulu-Natal, South Africa, we found a prevalence of schistosomiasis of 23%, showing the necessity of finding an accurate dose programme to be used in regular mass treatment. This study indicates a low accuracy of the WHO Tablet Pole as one in four girls would have received an insufficient dose of praziquantel (less than 30 mg/kg). Of the study population 35% were overweight or obese and this group was at particular risk of receiving an insufficient dose of praziquantel. Looking at primary school girls separately, a better performance of the WHO Tablet Pole was found; 89% would have received a dose above 30 mg/kg. The Modified Dose Pole would have indicated a right dose in 97% of all girls and was significantly more accurate than the WHO Tablet Pole, due to its ‘BMI correction’. When adding this correction to the WHO Tablet Pole, 94% of all girls would have received an appropriate dose.

Several previous studies have found that the WHO Tablet Pole is accurate, indicating a correct dose in more than 95% of the population [18,29,30]. The WHO Tablet Pole has therefore rightfully been recommended and used in mass-treatment of children, when no weight scales are available. However, since the previously mentioned studies were carried out, the global prevalence of overweight and obesity has continued to increase, affecting some countries more than others [24]. We found that overweight and obesity were very common in our study population, explaining the poor accuracy of the WHO Tablet Pole. The high prevalence of overweight and obesity in South Africa compared to neighbouring countries, has been indicated by the Global Burden of Disease Study [24]. Several studies investigating the accuracy of the WHO Tablet Pole in adult populations, have pointed out the issue of under-dosing of adults as height correlates poorly with bodyweight [22,23]. Our study shows the same in a young population. Yet under-dosing appears to pose a bigger problem among older children and adolescents than among younger primary school children.

We are aware that this research has limitations. Firstly, we did not investigate the accuracy of the WHO Tablet Pole in South African boys. The larger project from which our data was derived focuses on female genital schistosomiasis (FGS) as a risk factor for reproductive tract morbidity and HIV and did therefore not collect data on boys. We cautiously suggest that the management of schistosomiasis in girls might be more urgent as female genital schistosomiasis is suggested to cause an increased risk of HIV infection. However the findings on dosing praziquantel in overweight/obese individuals will likely also apply to boys as 19% of South African boys below the age of 20 years have been estimated to be either overweight or obese [24]. Hence the WHO Tablet Pole would indicate insufficient praziquantel dosages in boys as well.

This study solely focused on the delivery of the recommended praziquantel dose by different dose pole programmes. Further research is required to show if a dose pole programme which allows administration of low doses is less efficient in reducing the schistosomiasis prevalence on a population level. There is still some controversy surrounding the dose at which praziquantel should be administered, since effects of the drug have been reported at low doses of 20 mg/kg [16,31,32] However, low doses of praziquantel result in lower cure rates, which would limit the effect of treatment programmes on the overall morbidity and prevalence of schistosomiasis [16,32,33]. With regards to under-dosing overweight and obese individuals it should also be considered that the biological availability of the drug might be lower as a result of the drug distribution in body fat. We believe that, as long as no more data is available, treatment programmes should aim to deliver at least the recommended dose of 40 mg/kg [16,19].

Dosing by bodyweight remains the most accurate method to deliver the recommended dose of praziquantel. However, using weight scales would entail a substantial cost and logistical hurdle for implementing mass treatment programmes. When compared to dosing with a height pole, weighing each individual is more complicated and time consuming. In addition, weight scales are valuable, and safe storage and maintenance must be ensured either within each school or at each central point. In practice, the reliability of scales may be limited by incorrect use, defects in the calibration, or by lack of batteries. Dosing by height with a dose pole would represent a major alleviation of these inconveniences. Our study shows that the accuracy of the commonly used WHO Tablet Pole will be improved with a correction for overweight/obesity. In our study, this correction was based on the calculated BMI as the height and weight of each individual were measured. In practice this correction would be based on the dispenser’s estimation of the individual’s BMI since weight scales are not available. Based on a person’s physical appearance, one extra tablet of praziquantel could be administered to those who appear to be overweight/obese.

Dosing medication based on a person’s physical appearance was investigated by Alexander et al. for the dosing of invermectin [34]. An assistant was instructed to place people in one of four dosing categories based on their physical appearance. Alexander et al. showed that in 80% of the cases the estimation of the category was correct, with a maximum of one category off-target [34]. Taking that into account, it will likely be possible for fieldworkers to estimate if a person is overweight/obese and thus in need of an additional tablet. However, this method has not yet been validated for this specific context and would need further investigation for its accuracy. To achieve a more precise estimation of a person’s BMI, the use of pictograms has been suggested and this method was explored in a Danish population by Stunkard et al. [35]. The pictograms that were developed were later assessed in an Iranian population and were shown to be valid for discriminating between normal, overweight and obese people [36]. To our knowledge the use of pictograms to estimate BMI has not yet been validated in a South African population and therefore further research into this topic is needed before implementation of such a tool.

The WHO Tablet Pole remains an important tool in current dosing practices and is rather accurate in young children. However, to increase its accuracy for older age groups, incorporation of the additional height intervals of the Modified Dose Pole should be considered in future revision of the WHO Tablet Pole.

Our study suggests that in South Africa and other countries heavily affected by the obesity epidemic, the accuracy of anti-schistosomal treatment could be improved by using the WHO Tablet Pole with a correction for BMI by instructing fieldworkers to administer one extra tablet of praziquantel to those who appear overweight or obese.

## Supporting Information

S1 Table International Obesity Task Force (IOTF) body mass index cut-off points for underweight, overweight and obesity in girls (<18 years of age) [26]. (DOCX)

S1 Dataset (XLSX)

S2 Dataset (XLSX)
