# Supplementary material for: The Accuracy of Praziquantel Dose Poles for Mass Treatment of Schistosomiasis in School Girls in KwaZulu-Natal, South Africa
Source: PLoS Negl Trop Dis. 2016 May 3;10(5):e0004623. doi: 10.1371/journal.pntd.0004623 (PMC4854411; doi:10.1371/journal.pntd.0004623)
Supplement: S1 Table — (DOCX) [file pntd.0004623.s001.docx]

**Table S1: International Obesity Task Force (IOTF) body-mass index cut-off points for underweight, overweight and obesity in girls (<18 years of age) [26].**

| **Age (in years)** | **Underweight** | **Overweight** | **Obese** |
| --- | --- | --- | --- |
|  | *(corresponding with BMI 18.5 at age 18 years)* | *(corresponding with BMI 25 at age 18 years)* | *(corresponding with BMI 30 at age 18 years)* |
| 10.5 | 14.78 | 20.21 | 24.62 |
| 11.5 | 15.3 | 21.12 | 25.87 |
| 12.5 | 15.91 | 22.05 | 27.05 |
| 16.5 | 18.08 | 24.53 | 29.55 |
| 17.5 | 18.38 | 24.85 | 29.85 |
| Note: for each age group the cut-off point that corresponded with the middle of the specific age year was used | | | |
